# The use of convalescent plasma therapy in the management of a pregnant woman with COVID-19: a case report

**DOI:** 10.31744/einstein_journal/2022RC6550

**Published:** 2022-02-02

**Authors:** Lígia Walter Moura, Adolfo Wenjaw Liao, Romulo Negrini, Eduardo Zlotnik

**Affiliations:** 1 Hospital Israelita Albert Einstein São Paulo SP Brazil Hospital Israelita Albert Einstein, São Paulo, SP, Brazil.

**Keywords:** Coronavirus infections, COVID-19, Plasma, Convalescent plasma, Pregnancy, Pregnancy complications, infectious, Antibodies, neutralizing

## Abstract

The coronavirus disease 19 (COVID-19) is responsible for the current worldwide pandemic. Treatment and prophylaxis are still under investigation. Convalescent plasma therapy could be an alternative. We report a case of a 41-year-old patient, at 28 weeks of gestation, was hospitalized with COVID-19. On the 10^th^ day after onset of symptoms, the clinical picture worsened, and she required high-flow oxygen therapy (30L/minute), with 92% oxygen saturation, and chest X-ray showing mild bilateral opacities at lung bases. Blood tests showed D-dimer 1,004ng/mL, C-reactive protein 81mg/L, pro-calcitonin 0.05ng/mL and interleukine-6 42.9pg/mL. The therapy chosen was Tazocin^®^ 12g/day, vancomycin 2g/day, and methylprednisolone 40mg/day. In addition, convalescent plasma therapy was administered (275mL) uneventfully, including SARS-CoV-2 antibodies and neutralizing antibodies >1:160. The patient had a fast recovery. The early administration of convalescent plasma, with high titers of neutralizing antibodies, may be an alternative option for severe COVID-19 during pregnancy, until further studies demonstrate an efficient and safe treatment or prophylaxis.

## INTRODUCTION

Severe acute respiratory syndrome coronavirus 2 (SARS-CoV-2) is the enveloped ribonucleic acid (RNA) virus, which causes the current outbreak of coronavirus disease 2019 (COVID-19), first reported in China, in December 2019.^(1,2)^ Currently, no specific treatment is available.

The World Health Organization (WHO) has supported many clinical trials using drugs such as remdesivir, chloroquine/hydroxychloroquine, lopinavir and ritonavir. Most of them have revealed discouraging results.^(3-7)^

Investigations about treatment and prophylaxis involving convalescent plasma have been approved by the Food and Drug Administration (FDA), and 135 studies had been registered at the National Library of Medicine (NLM) of the United States.^(8,9)^ The first use of convalescent plasma in a viral infection was in the Spanish influenza pandemic (1918-1920), and later tested during the Ebola epidemic, in West Africa (2013-2016). Promising results were also obtained for treatment of infections due to the Middle East respiratory syndrome coronavirus (MERS-CoV), severe acute respiratory syndrome coronavirus 19 and H5N1.^(8)^

Based on the passive immunization hypothesis, convalescent plasma has been used as an additional intervention for critically-ill patients with COVID-19 infection.^(2)^ Although there are 147 ongoing clinical trials using convalescent plasma in COVID-19 patients, there is a lack of knowledge in the obstetrical population, since only three studies included pregnant women.^(10)^

This report presents a case of COVID-19 infection during pregnancy treated with convalescent plasma.

This study was approved by the Ethics Committee of *Hospital Israelita Albert Einstein* (approval number: 4.287.295; CAAE: 35391920.7.0000.0071). In addition, it is stated that the patient signed the Informed Consent Form.

## CASE REPORT

A 41-year-old female patient, with no comorbidities, cough or fever, presented vomiting, diarrhea, weakness, headaches, anosmia, muscle and joint pain, at 28 weeks of pregnancy.

On the 7^th^ day after onset of symptoms, a nasopharyngeal secretion real time reverse transcription polymerase chain reaction (rRT-PCR) test confirming SARS-CoV-2 infection, and she was admitted to the hospital. Upon physical examination, blood pressure of 99x64mmHg, 97% oxygen saturation (SpO_2_), decreased breath sounds on the right lung base, and back pain that was movement-dependent.

Initial laboratory workup included respiratory pathogens panel testing, confirming of SARS-CoV-2, and positive rRT-PCR. Blood tests also showed an increased D-dimer (616ng/mL FEU), C-reactive protein (CRP; 13mg/L), and normal kidney and liver functions.

The patient was prescribed ceftriaxone 2g/day, azithromycin 1g/day, and low-molecular heparin 40mg/day. Azithromycin was discontinued soon after the first dose (500mg) due to intolerance and vomiting.

**Figure 1:**
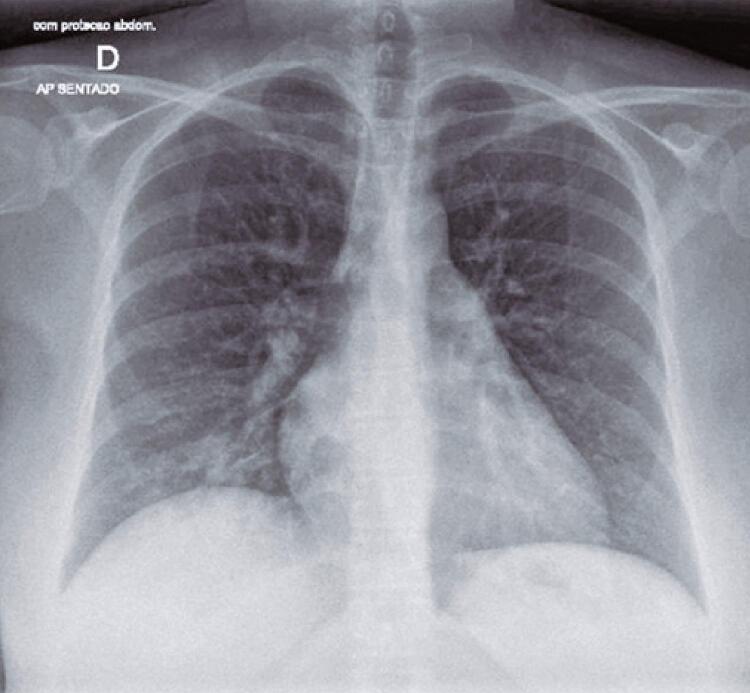
Anteroposterior chest X-ray with interstitial involvement and mild opacity at the right lung base

On the following day, the patient was breathing normally (SpO_2_ 95% to 97%), still complaining of weakness, joint pain, diarrhea, and the same pulmonary auscultation. Ultrasound confirmed normal fetal growth and well-being, and no abnormalities on lower limbs Doppler scan. Lung ultrasound and chest X-ray showed interstitial involvement and mild opacity at the right lung base, respectively (Figure 1). Gastroenteritis panel investigation was negative.

Clinical deterioration was observed on the 9^th^day, including chest pain and dyspnea (SpO_2_ 88% to 91%), and required nasal oxygen catheter at 1L/minute. Breath sound was diminished bilaterally up to the middle third, with associated atypical adventitious sounds on pulmonary auscultation. Mild opacities on lung bases were seen bilaterally on repeated chest X-ray, and blood tests showed an increase in D-dimer (878ng/mL FEU) and CRP (66mg/L) levels. Maternal echocardiography did not show abnormalities and electrocardiogram demonstrated final delay in ventricular conduction and low voltage QRS complex in precordial leads.

On the 10^th^day, the patient presented shallow breathing, use of accessory muscles, atypical bilaterally adventitious sounds up to the pulmonary apex on auscultation, and SpO_2_ of 92%. High-flow oxygen therapy (30L/minute) was then initiated. Ceftriaxone was discontinued and she was initiated on Tazocin^®^ 12g/day, vancomycin 2g/day, and methylprednisolone 40mg/day. Blood tests showed D-dimer level of 1,004ng/mL, CRP 81mg/L, pro-calcitonin 0.05ng/mL, and interleukine-6 (IL-6) 42.9pg/mL.

A multidisciplinary team decided not to prescribe chloroquine, due to electrocardiogram findings, and antivirals, because FDA had not approved these drugs for COVID-19. Convalescent plasma therapy was brought up as an alternative due to the lack of severe side effects and considering the case as “in potential need”, according to FDA classification. The couple gave consent for convalescent plasma therapy after being informed about potential risks and benefits.

The convalescent plasma was administered (275mL) uneventfully, and included SARS-CoV-2 antibodies and neutralizing antibodies >1:160. Despite persistent diarrhea, a quick improvement in respiratory pattern was observed 12 hours later, adventitious sounds were auscultated up to the middle third of both lungs and vancomycin was discontinued; nasal oxygen catheter flow was decreased to 1 to 2L/minute to maintain SpO_2_ at 95%. Clinical improvement was observed throughout the following 3 days, breaths sounds remained bilaterally diminished on lung bases, and reduced D-dimer (863ng/mL) and CRP (9.8mg/L) levels were observed.

After the 21^th^day, the patient was breathing environment air with SpO_2_ at 95%, with no symptoms. A new obstetric ultrasound demonstrated adequate fetal growth and Dopplervelocimetry at 30 weeks of pregnancy. At this point she was discharged, and followed up in prenatal care appointments. At 34 weeks an ultrasound was requested showing polyhydramnios, which concerned the obstetrician, and a new ultrasound was repeated at 36 weeks and 6 days, showing oligohydramnios (14mm). The newborn birth weight was 2,750g, Apgar 8/9, with meconium and no amniotic fluid.

## DISCUSSION

To the best of our knowledge, this is the fifth case report involving use of convalescent plasma for the treatment of COVID-19 infection during pregnancy. Grisolia et al.,^(9)^Anderson et al.,^(1)^and Soleimani et al.,^(10)^case reports showed patients during the second trimester of pregnancy, who improved after using convalescent plasma with antibiotics and antivirals. The patients described by Grisolia et al.,^(9)^and Soleimani et al.,^(10)^ were obese, but did not require mechanical ventilation. The patient reported by Anderson et al.,^(1)^was obese, had asthma and type 2 diabetes, and needed mechanical ventilation. Zhang et al.,^(11)^described a third-trimester pregnant woman with no comorbidities, who developed a secondary bacterial infection and complications due to intrauterine death; in this case, convalescent plasma was used after cesarean section.

Physiological cardiovascular and respiratory changes make pregnant women susceptible to severe pneumonia, with increased morbidity and mortality. The majority of pregnant women infected with COVID-19 are asymptomatic, but the disease can be severe and fatal in some of them, particularly during the second and third trimesters.^(12-14)^ Showed that 31.4% (22 out of 70) of admissions were in the intensive care unit. Mechanical ventilation was used in 23.2% (16 of 69) and maternal death rate was 11.4% (nine of 79).^(12)^ Most of pregnancy specific protocols used empiric broad-spectrum antibiotics therapy upon admission, and our hospital´s protocol suggests ceftriaxone and azithromycin as empiric antibiotics when a COVID-19 patient is hospitalized, with possibility of escalation.^(14-16)^

It has been demonstrated that SARS-CoV-2 binds to target cells through a homotrimeric spike (S) glycoprotein specific to angiotensin-converting enzyme 2 (ACE2) receptor.^(17)^ Although, there is a lack of knowledge regarding the pathophysiology involved in COVID-19 infection and specific treatment, different protocols have emerged and several trials are being carried out.^(1)^

Administration of convalescent plasma is one of the strategies proposed and its mechanism involves reducing viraemia and the inflammatory response, by administering neutralizing antibodies to prevent the entry of SARS-CoV-2 in the host cells, by blocking S protein and ACE2 receptor binding.^(17,18)^ Other plasma compounds, such as anti-inflammatory cytokines, clotting factors, natural antibodies, defensins, pentraxins and other substances, may also contribute to immunomodulation. In our case, the clinical improvement after convalescent plasma therapy was demonstrated by a rapid increase in SpO_2_ and decrease in CRP levels.^(17,19)^

SARS-CoV-2 viral neutralization in lung tissue and blood circulation depends on two important factors: antibody titers, which must be greater than 1:80,^(11,18,19)^ and start during the first 14 days. However, the precise best moment for administration is still not clear.^(11)^

## CONCLUSION

Since pregnancy is an exclusion criterion in most trials, the present case demonstrated early administration of convalescent plasma, with high titers of neutralizing antibodies, may be an option for severe COVID-19 infection during pregnancy, until further studies demonstrate an efficient and safe treatment or prophylaxis. Another important aspect in this case report was to find polyhydramnios, followed by oligohydramnios and meconium, after COVID-19, demonstrating further studies should be carried out regarding the gestational outcome.
